# Unmyelinated White Matter Loss in the Preterm Brain Is Associated with Early Increased Levels of End-Tidal Carbon Monoxide

**DOI:** 10.1371/journal.pone.0089061

**Published:** 2014-03-12

**Authors:** Cornelie A. Blok, Karina J. Kersbergen, Niek E. van der Aa, Britt J. van Kooij, Petronella Anbeek, Ivana Isgum, Linda S. de Vries, Tannette G. Krediet, Floris Groenendaal, Hendrik J. Vreman, Frank van Bel, Manon J. Benders

**Affiliations:** 1 Department of Neonatology, Wilhelmina Children's Hospital, Utrecht, The Netherlands; 2 Image Sciences Institute, University Medical Center, Utrecht, The Netherlands; 3 Department of Pediatrics, Stanford University School of Medicine, Stanford, California, United States of America; The University of Melbourne, Australia

## Abstract

**Objective:**

Increased levels of end-tidal carbon monoxide (ETCOc) in preterm infants during the first day of life are associated with oxidative stress, inflammatory processes and adverse neurodevelopmental outcome at 2 years of age. Therefore, we hypothesized that early ETCOc levels may also be associated with impaired growth of unmyelinated cerebral white matter.

**Methods:**

From a cohort of 156 extremely and very preterm infants in which ETCOc was determined within 24 h after birth, in 36 infants 3D-MRI was performed at term-equivalent age to assess cerebral tissue volumes of important brain regions.

**Results:**

Linear regression analysis between cerebral ventricular volume, unmyelinated white matter/total brain volume-, and cortical grey matter/total brain volume-ratio and ETCOc showed a positive, negative and positive correlation, respectively. Multivariable analyses showed that solely ETCOc was positively related to cerebral ventricular volume and cortical grey matter/total brain volume ratio, and that solely ETCOc was inversely related to the unmyelinated white matter/total brain volume ratio, suggesting that increased levels of ETCOc, associated with oxidative stress and inflammation, were related with impaired growth of unmyelinated white matter.

**Conclusion:**

Increased values of ETCOc, measured within the first 24 hours of life may be indicative of oxidative stress and inflammation in the immediate perinatal period, resulting in impaired growth of the vulnerable unmyelinated white matter of the preterm brain.

## Introduction

Carbon monoxide (CO), a product of oxidative degradation of heme, diffuses from the vascular compartment to alveolar air, where it can be quantitated in exhaled air as end-tidal CO (ETCOc) [1]. ETCOc can be elevated during episodes of oxidative stress and inflammation through increased expression of inducible heme oxygenase (HO-1) [1]–[3]. In preterm infants with severe respiratory distress syndrome subsequently developing bronchopulmonary dysplasia (BPD) and in infants of preeclamptic mothers with HELLP syndrome we indeed found an association between elevated ETCOc and oxidative stress and inflammation using malondialdehyde and pro-inflammatory cytokines as markers [4], [5].

In up to 50% of extremely and very preterm infants (subtle) white matter damage (WMD) is the most important cause of neuronal-axonal disease with subsequent adverse cognitive, behavioral and neuromotor outcome [4]. Perinatal inflammation and hypoxia-ischemia are suggested to play a causative role [6]–[9]. WMD is characterized by a marked decrease in immature oligodendrocytes, which are extremely sensitive to reactive oxygen and nitrogen species and pro-inflammatory cytokines, leading to inadequate myelinization and white matter axon formation [10], [11]. In a recent study in preterm infants we reported a relation between elevated ETCOc levels during the first 24 hours of life and adverse neurodevelopmental outcome at 3.5 years of age [12].

In the present study we aimed to investigate the relation between ETCOc during the first 24 hours of life, and white matter volume, as measured by 3D volumetric MRI at term equivalent age in extremely and very preterm infants.

## Methods

ETCOc was determined within 24 hours of life in a cohort of 156 preterm infants with gestational age (GA) of less than 32 weeks, consecutively admitted to the Neonatal Intensive Care Unit of the Wilhelmina Children's Hospital, University Medical Center Utrecht from August 2007 to September 2009. In 36 of these infants 3D volumetric MRI was obtained at term equivalent age for determination of various cerebral tissue classes. In 30 infants the MRI was routinely performed being clinical practice in our NICU in infants with GA 28 weeks or less. In 6 other infants intracranial pathology was suspected clinically. Neonatal and obstetrical data were collected prospectively.

### 

#### Ethics statement

The study was reviewed and approved by the Institutional Review Board of the University Medical Center Utrecht. Parents of included infants provided written informed consent.

Periventricular-intraventricular hemorrhage (PIVH) was graded according to the grading of Papile et al [13]: grade I, blood in the germinal matrix; grade II, blood in the lateral ventricle(s) without acute distension of the ventricle(s); grade III, blood in the lateral ventricle(s) with acute distension of the ventricle(s); grade IV, venous hemorrhagic infarction.

Diagnosis of severity of infant respiratory distress syndrome (RDS) was based on clinical symptoms and radiographic signs as defined by Giedion *et al* [14]: without (moderate RDS) or with (severe RDS) the need for surfactant replacement therapy. Decisions on surfactant replacement therapy were made by the attending neonatologist and were based on a defined protocol used in our unit.

Arterial blood pressure was measured continuously via indwelling arterial catheter. To assess the intensity of blood pressure support in infants with hypotension, a “blood pressure support scoring system” was used as described earlier [15]: Score 1: volume expansion and/or dopamine ≤5 µg/kg/min; Score 2: dopamine >5 ≤10 µg/kg/min; Score 3: dopamine >10 µg/kg/min, or dopamine and dobutamine ≤10 µg/kg/min; Score 4: dopamine and dobutamine >10 µg/kg/min; and Score 5: additional adrenalin and/or corticosteroids.

### Measurement of ETCOc

Endogenously produced carbon monoxide (CO), usually quantitated as ETCOc, was measured within the first 24 hours of age, using the COCO2 Puff Analyzer (Everest Biomedical Instruments, Chesterfield, USA) as previously described [4], [12]. Briefly, this non-invasive bedside instrument uses electrochemical sensors for measurement of CO and hydrogen and an infrared optical bench for measurement of end-tidal carbon dioxide (ETCO_2_). Because excessive concentrations of hydrogen (H_2_) interfere with accurate CO measurements, the instrument will not permit CO measurements when breath H_2_ concentrations exceed 50 parts per million (ppm) or µL/L. CO_2_ measurements and breath rate measurements can be used by the clinician to evaluate the quality of sampling. This instrument has a range of 0.0 to 25.0 ppm CO with a resolution of 0.1 ppm. Its accuracy is 0.3 ppm or 10% of the reading (whichever is greater). A single use, flexible nasal sampler (1.5 mm O.D.) was connected to the analyzer. It was then inserted approximately 5 mm into the nostril when infants were breathing spontaneously and/or on continuous positive airway pressure (CPAP) or, in case of mechanical ventilation, inserted into the proximal part of the endotracheal tube via a T-connector. During 90 s breathing or artificial ventilation, expired air is continuously sampled for determination of ETCO and ETCO_2_. At completion of the procedure the sampler was disconnected from the device and the CO-concentration in room air was determined and used to correct for CO (COc) in the inhaled air (typically 0.5 ppm). In case of mechanical ventilation, CO in room air was used as a substitute for correction of inhaled CO. Using the COCO2 Puff Analyzer, ETCOc can be measured reliably and reproducibly even in tiny ventilated infants [4], [12], [16]. Measurements were performed in duplicate and the mean value of the two measurements was reported. ETCOc was expressed as ppm (µL/L). Measurements rejected by the COCO2 Puff Analyzer due to excessive levels of hydrogen in the exhaled air were excluded.

### Volumetric MRI Imaging

All MR investigations were performed at term equivalent age on a 3.0 Tesla MR system (Philips Healthcare, Best, The Netherlands) using a sense head coil. The infants were sedated with 50–60 mg/kg chloralhydrate by gastric tube 15 minutes prior to the examination. During MR examination, the infants were placed in a vacuum fixation pillow to reduce movement and hearing protection was administered while heart rate, transcutaneous oxygen saturation and respiratory rate were monitored. A neonatologist was present throughout the procedure.

The protocol involved conventional axial acquisitions: 1) axial 3D T1-weighted: TR = 9.4 ms; TE = 4.6 ms; flip angle = 8 degrees, scan time = 3.44 min, FOV = 180×180; reconstruction matrix = 512×512; consecutive sections with slide thickness = 2.0 mm with no gap; number of sections = 50, in-plane resolution 0.35 mm×0.35 mm. and 2) axial T2-weighted images: TR = 6293 ms; TE = 120 ms; scan time = 5.40 min; FOV = 180×180; reconstruction matrix = 512×512; consecutive sections with slice thickness = 2.0 mm with no gap; number of sections = 50, in plane resolution 0.35 mm×0.35 mm. The axial T1- and T2-weighted images were used for segmentation of the different brain structures.

Part of the infants was scanned in the coronal plane. Coronal acquisitions were 1) coronal 3DT1-weighted: TR = 9.5 ms; TE = 4.6 ms; flip angle =  8 degrees, scan time = 7.02 min, FOV = 200×200; reconstruction matrix = 256×256; consecutive sections with thickness = 1.2 mm; number of sections = 110, in-plane resolution 0.78 mm×0.78 mm and 2) coronal T2-weighted images: TR = 4847 ms; TE = 150 ms; scan time = 5.05 min; FOV = 180×180; reconstruction matrix = 512×512; consecutive sections with thickness = 1.2 mm; number of sections = 110, in-plane resolution 0.35×0.35 mm. These images were converted to the axial plane before segmentation was performed.

We used our extended user-independent, fully automatic neonatal brain segmentation method to estimate volumes of eight different brain structures: cortical gray matter (cGM), deep gray matter (basal ganglia and thalamus), cerebral ventricle (cerV)-volume, cerebrospinal fluid, myelinated white matter (MWM), unmyelinated (Unm)WM, brainstem and cerebellum [17]–[19]. Voxels were classified by the k-Nearest Neighbor classification system and based on their signal intensities on the T1- and T2- weighted images and their x-, y- and z-coordinates. For each voxel the probability for each tissue type was calculated, which was defined as the chance that the voxel belonged to each of the eight tissue types. The various volumes were determined by adding up the probabilities of all voxels for each tissue type multiplied by the voxel volume. All slices were segmented manually into the eight tissue classes; the result was used as ‘gold standard’. The Dice similarity indices (Dice SI) were between 0.78–0.93 except for the MWM (Dice SI 0.57).

**Figure 1:**
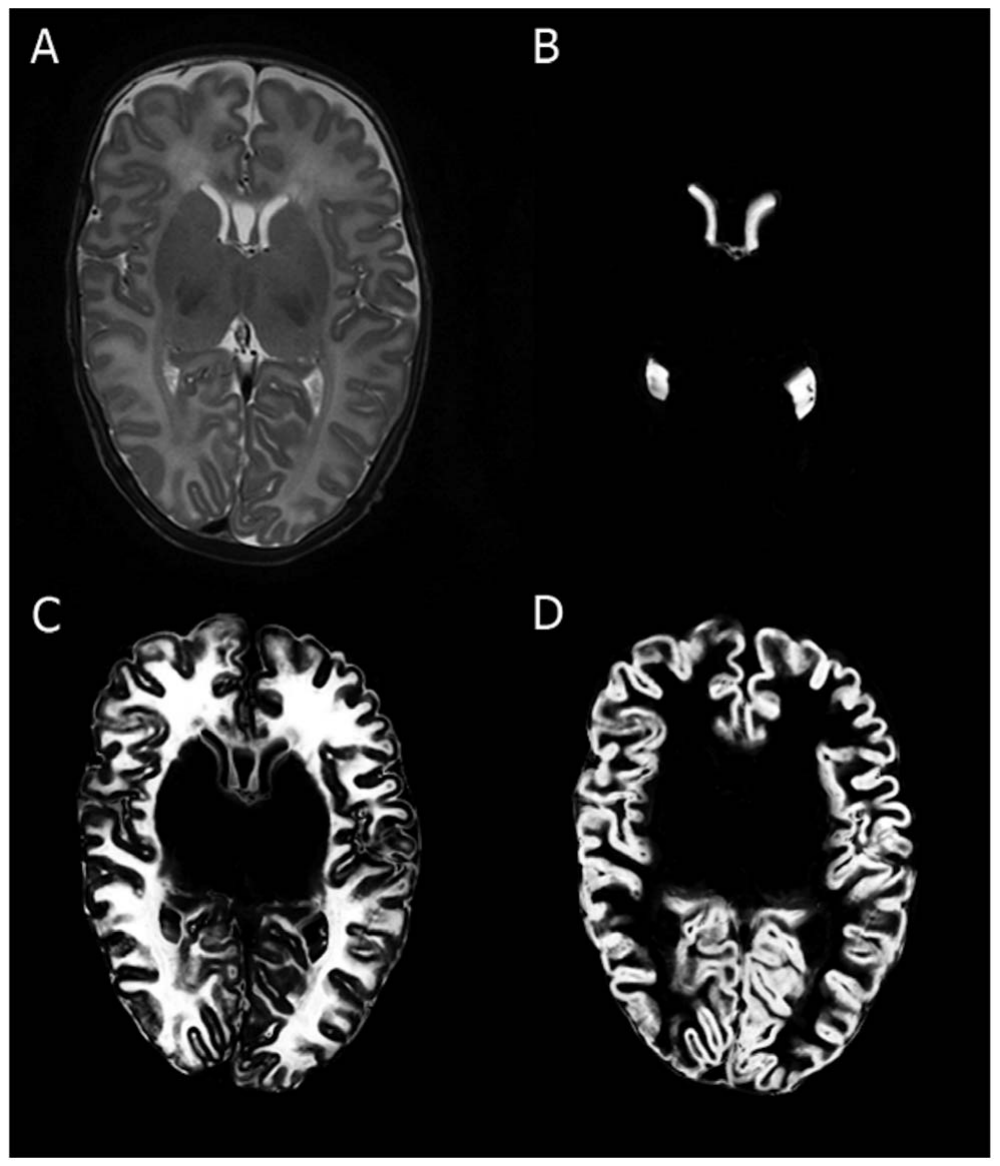
Representative example of a T2-weighted scan (fig. 2A) with segmentations of cerebral ventricles (fig. 2B); unmyelinated white matter (fig. 2C); and cortical grey matter (fig. 2D).

We tested for the difference in volume in a subgroup of 5 patients scanned with both coronal and axial acquired images and we did not find any differences in volumes (paired t-tests: for all tissue classes p>0.2; specifically for UnmWM 0.166, cGM 0.371, TBV 0.783). Furthermore, visual inspection was performed of all images. This allowed us to combine these sets into one cohort (see method described by Kersbergen et al [19]). Furthermore, our method performs accurately since it was validated on its performance with respect to a reference standard of manual segmentations (http://neobrains12.isi.uu.nl/mainResults_Set1.php). The dice coefficient for cortical gray matter was 0.83 (the best method had 0.85) and for unmyelinated white matter it was 0.88 (the best method had 0.91). The performance in comparison with a total of 6 neonatal brain segmentation programs (3T) was rather well (http://neobrains12.isi.uu.nl; paper is under review). In the present study the analysis was restricted to cerV-volume, UnmWM and cGM (see figure 1A–D).

### Statistical analysis

Data are summarized as means ±1 SD or median and ranges where appropriate. The temporal relationship between ETCOc, being an indicator of oxidative stress and perinatal inflammation, and the different tissue classes was investigated using simple linear regression analysis. cGM and/or UnmWM volumes were corrected for total brain volume (TBV) using the ratio between cGM or UnmWM respectively and TBV.

Multivariable Linear Regression (MLR) analysis was then used to investigate the relationship between the volumes (or ratios) of relevant MRI-measured brain regions such as cerV-volume, GM-TBV ratio and the UnmWM-TBV ratio, and ETCOc, postmenstrual age at MRI (PMA) and 2 conditions which may have a negative effect on brain growth (grade of PIVH and culture proven sepsis [17], [20]). Three MLR models were then constructed with cerV-volume (1), UnmWM-TBV ratio ratio (2) or cGM-TBV (3) as the respective dependent variables, and ETCOc, PMA at MRI, PIVH grading and sepsis as independent variables. We repeated these MLR analyses with the same independent variables, but replaced the dependent variable by yes/no BPD. This was done because of the earlier established relation between elevated ETCOc and the subsequent occurrence of BPD [4], [16].

Bilirubin production (being an indicator of hemolysis) [20], maternal smoking [21] and perinatal sepsis [22] can all exert an effect on endogenous CO-production. To investigate whether or not these factors confound the relationship between ETCOc and the volumes (or ratios) of relevant MRI-measured brain regions (cerV-volume, GM-TBV ratio and the UnmWM-TBV ratio), MLR analysis was repeated with ETCOc as dependent variable and maximal serum bilirubin concentration in the first 48 h of life, maternal smoking (yes/no) and perinatal sepsis (yes/no) as independent variables. Results were presented as coefficients of the independent variables with the 95% confidence intervals (CI). The final model was reduced to include only the significant variables at the 0.05 level. In addition, adjusted R2 of the model was presented.

For statistical analysis Statview© II was used (Abacus Concepts, Inc., Berkeley, CA, USA). *P*<0.05 was considered statistically significant.

## Results

The most important clinical characteristics of the 36 studied infants are shown in table 1. GA ranged from 25.0 to 31.7 weeks (median 27.0 weeks), birth weight ranged from 680 to 2000 grams (median 951 grams) and PMA at MRI ranged from 40.2 to 43.3 weeks (median 41.1 weeks). ETCOc ranged from 0.7 to 5.5 ppm (median 2.2 ppm); ETCO_2_ ranged from 2.7 to 7.2 ppm (median 4.4 ppm). In none of the infants we detected (too) high breath H_2_ concentrations. Hemoglobin and hematocrit values were always in the normal range (median [ranges]: 10.2 [7.8-to-12.2 mmol/L] and 0.49 [0.37-to-0.59 l/l] respectively), as were pCO2 values during measurement of ETCOc (median [ranges]: 41[34–54] mmHg). Maximal plasma bilirubin concentrations during the first 48 hours of life ranged from 50 to 210 mmol/L, median value 114 mmol/L.

**Table 1:** Important clinical characteristics of the 36 infants.

Gestational age (weeks) (mean ± SD)	27.2±1.7
Birth weight (grams) (mean ± SD)	1027±47
Postmenstrual age at MRI (weeks) (mean ± SD)	41.4±0.8
Pre-eclampsia (n)	3
Maternal Smoking (n)	6
Mode of Delivery (n: Vaginal/Cesarean Section)	15/21
Apgar score at 5 minutes median [range])	8 [4–10]
Maximal serum (Total) Bilirubin conc (mmol/L) first 48 hours (median[range])	114 [50–210]
Infant respiratory distress syndrome (n)	
No	14
Moderate	14
Severe	8
Bronchopulmonary dysplasia (n)	15
Periventricular-Intraventricular hemorrhage (n)	
No	22
Grade I	5
Grade II	2
Grade III	4
Grade IV	3
Gender (n): Male/Female	16/20
Hemodynamically important Ductus Arteriosus (n)	10
Maximal Blood Pressure Support Score (n);	
No support	12
Score 1	8
Score 2	8
Score 3	2
Score 4	0
Score 5	6
Culture proven sepsis (n)	
No	25
Yes	11
Perinatally (at admission)	6

Table 2 provides the MRI-determined volumes (means ± SD and ranges) in mL of the various brain regions.

**Table 2:** MRI-measured volumes in mL (means ± SD and ranges) of selected brain regions of the 36 infants.

Cerebral Ventricular-volume	11.1±5.6 [3–28.9]
Unmyelinated White Matter	161±21 [95–195]
Myelinated White Matter	2.80±1.00 [0.9–5.3]
Cortical Gray Matter	167±23 [116–218]
Total Brain Volume	391±34 [294–452]
Basal Ganglia/Thalamus	22.5±2.8 [14.0–27.3]
Cerebral Spinal Fluid	96±19 [59–140]

### Relation between ETCOc and MRI-measured volumes of selected brain regions

**Figure 2:**
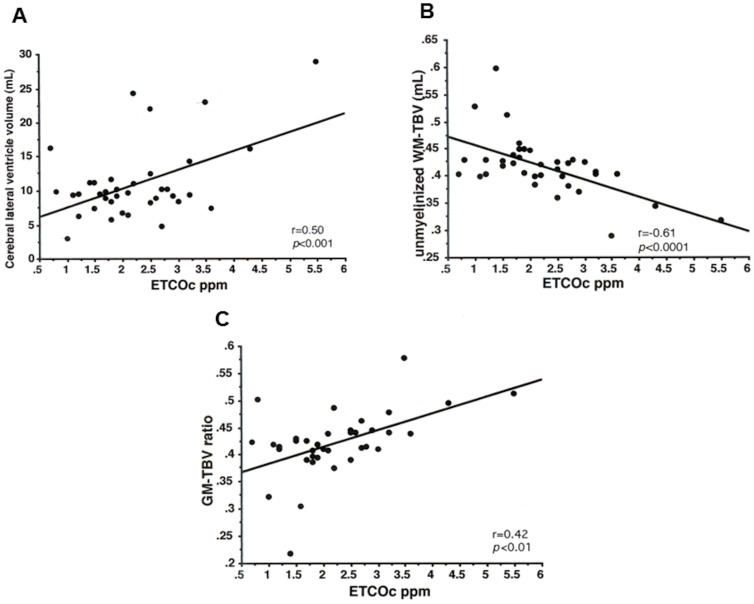
Individual regression plots between ETCOc (X-axis) and cerebral ventricle volume (mL) (fig. 1A); unmyelinated white matter-total brain volume (unmyelinated WM-TBV) ratio (fig. 1B); and cortical grey matter-total brain volume (cGM-TBV) ratio respectively (fig. 1C).

Simple linear regression analysis showed significant correlations of ETCOc with cerV-volume (r = 0.51, *p*<0.01), UnmWM-volume (r = −0.53, *p*<0.001); UnmWM-TBV ratio (r = −0.63, *p*<0.0001); cGM-volume (r = 0.41, *p*<0.01); and cGM-TBV ratio (r = 0.53, *p*<0.001). Plots of the most important regression analyses are shown in figure 2 A–C.

Multivariable analysis of cerV-volume revealed a positive relationship between ETCOc and cerV-volume with an overall increase of ventricular volume of 3.0 mL per 1 ppm of ETCOc (95% CI: 1.20–4.72) and with PIVH (95% CI: 0.25–7.28). PMA, BPSS or sepsis had no significant effects on cerV-volume.

Multivariable analysis of UnmWM-TBV ratio demonstrated a reverse relationship between ETCOc and UnmWM-TBV ratio with an overall decrease of the UnmWM-TBV ratio of 0.03 per 1 ppm of ETCOc (95% CI: −0.42–(−) 0.15). PIVH, PMA, or sepsis showed no significant effect on the UnmWM-TBV ratio.

Multivariable analysis of the cGM-TBV ratio revealed a positive relationship between ETCOc and cGM-TBV ratio with an overall increase of the cGM-TBV ratio of 0.03 per 1 ppm of ETCOc (95%CI: 0.01–0.04). Here too, PIVH, PMA, or sepsis had no significant independent effect on the cGM-TBV ratio.

In this cohort of 36 infants multivariable analysis did not reveal an effect of ETCOc, PIVH, PMA or sepsis on the subsequent occurrence of BPD.

Multivariable analysis showed no effect of maternal smoking, perinatal sepsis or hemolysis (as indicated by serum bilirubin concentration) on ETCOc.

## Discussion

1) The positive correlation between ETCOc levels at birth and the volume of the cerebral ventricles; 2) the negative correlation between ETCOc and the UnmWM-TBV ratio; and 3) the results of the multiple linear regression, where ETCOc appeared to be the only significant independent variable in both equations among other independent factors which may also be related with loss of WM [21]–[25], may suggest a relationship between perinatal oxidative stress and inflammation and loss of unmyelinated WM, assuming that ETCOc may be used as an indicator of oxidative stress and inflammation (1–3). Indeed, in earlier studies in individuals with asthma a strong association was reported between raised levels of exhaled CO and severity of pulmonary disease with an increased expression of heme oxygenase-1 in airway macrophages, interpreted to be due to oxidative stress [3], [26]. In addition, other studies reported a relation between pulmonary disease and exhaled carbon monoxide [27]. In an earlier study of our group an association was found between severe respiratory distress syndrome and subsequent development of BPD and increased ETCOc values [4]. Additionally, in a study in premature infants of pre-eclamptic mothers with HELLP syndrome we found evidence for increased non-enzymatic lipid peroxidation, as indicated by increased levels of malondialdehyde and the pro-inflammatory cytokine IL-8, which coincided with increased ETCOc levels [5].

Despite the quite strong association between an elevated ETCOc-value and white matter damage (particularly indicated by the decreased UnmWM-TBV ratio), multivariable analysis revealed no additional effect of maternal smoking, perinatal sepsis or hemolysis, as indicated by serum bilirubin concentration, on ETCOc. For maternal smoking and perinatal sepsis this may be due to the small number of infants with smoking mothers and perinatal sepsis in this cohort of infants. However, we have no plausible explanation for the lack of association between ETCOc and BPD, which is reported in several studies, including one of our group [4], [16].

That oxidative stress and pro-inflammatory cytokines are key-players in the infants of the present study with increased ETCOc levels and subsequent WM loss is supported by the results of an earlier study of our group, which showed, in a large cohort of very preterm born children (n = 105) that long-term adverse neurodevelopmental outcome was associated with increased endogenous CO production, whereas ETCOc values below 2.0 ppm during the first day of life indicated a favourable neurodevelopmental outcome [12]. This strongly suggests that there is a causal relationship between inflammation and oxidative stress, as indicated by the increased ETCOc, and brain damage.

However, one finding of the present study is intriguing: How should we interpret the positive relation between ETCOc and MRI-determined cGM volume? WMD is characterized by marked astrogliosis and microgliosis and initially by a decrease of pre (un)-myelinating oligodendrocytes, which are subsequently replaced by abnormal oligodendroglial progenitors which never reach full differentiation [6], [28], [29]. Moreover, (selective) neuronal loss in several cortical regions often occurs in WMD [28]. However, the layer of abnormal progenitor cells which do not reach the capacity to form myelin has been shown to be situated at the cortical grey-white matter boundaries [28], [30], which may change the intensity of the transition zone between cGM and WM, as is also reported in other abnormal cell patterning at the grey-white matter boundary [31], [32]. We can only speculate that in our cohort of very preterm infants an indistinct transition of GM-to-abnormal white matter in those infants with WMD has led to overestimation of the grey volume measurements. It is clear, however, that this assumption needs confirmation. Whether this finding could be explained by ill-defined GM/WM boundary, because of limited spatial resolution remains open. Nevertheless, the method seems to be accurate (19) and the ‘best-that-can-be-done- today’ scenario for our patient population.

We could not confirm that neonatal sepsis, in the present study mostly due to coagulase-negative staphylococci, was related to WM damage as reported repeatedly [33], [34]. Here too, the limited number of included patients in the present study may be an important reason for this lack of evidence, although in a larger prospective cohort study on coagulase-negative staphylococcal sepsis from our group we were unable to detect an association with WM damage [35].

The present study has indeed other limitations besides the small sample size. ETCOc is not a direct indicator of lipid peroxidation or pro-inflammation. On the other hand, as already stated above, we have previously shown that ETCOc correlated positively with malondialdehyde, increased pro-inflammatory cytokines and interleukins, which suggests that ETCOc can be used as an estimator of free radical stress and pro-inflammatory activity. Furthermore, we did not include myelinated WM in our analysis. However, myelinated WM is only a very small part of the WM at term equivalent age and shows much variation during a short period of time, making it difficult to obtain accurate measurements with our volumetric segmentation method. We therefore decided to exclude this brain structure from the analysis.

In conclusion, in the present study ETCOc was positively related to cerebral ventricle size and inversely to the unmyelinated white matter-total brain volume ratios, suggesting loss of (white matter) brain tissue. Perinatal inflammation and oxidative stress, as estimated by ETCOc, may be an important cause for this phenomenon, although future studies are mandatory to prove this postulation. Future studies on the usefulness of ETCOc measurements during the first 24 hours of life to predict cerebral white matter damage in the very preterm infant seem warranted.
